# Selected parameters of the corneal deformation in the Corvis tonometer

**DOI:** 10.1186/1475-925X-13-55

**Published:** 2014-05-03

**Authors:** Robert Koprowski, Anita Lyssek-Boron, Anna Nowinska, Edward Wylegala, Henryk Kasprzak, Zygmunt Wrobel

**Affiliations:** 1 Department of Biomedical Computer Systems, University of Silesia, Faculty of Computer Science and Materials Science, Institute of Computer Science, ul. Będzińska 39, Sosnowiec 41-200, Poland; 2 Ophthalmology Clinic, Medical University of Silesia, District Railway Hospital in Katowice, Katowice, Poland; 3 Institute of Physics, Wroclaw University of Technology, Wybrzeze Wyspianskiego 27, Wroclaw 50-370, Poland

**Keywords:** Eye biomechanics, Corvis tonometer, Image processing, Measurement Automation, Segmentation

## Abstract

**Introduction:**

Contemporary ophthalmology knows many methods of measuring intraocular pressure, namely the methods of non-contact and impression applanation tonometry. In non-contact applanation tonometers, e.g. the Corvis, the corneal flattening is caused by an air puff. Image registration of the corneal deflection performed by a tonometer enables to determine other interesting biomechanical parameters of the eye, which are not available in the tonometer. The measurement of new selected parameters is presented in this paper.

**Material and method:**

Images with an *M* × *N* × *I* resolution of 200 × 576 × 140 pixels were acquired from the Corvis device in the source recording format *.cst. A total of 13'400 2D images of patients examined routinely in the Clinical Department of Ophthalmology, in District Railway Hospital in Katowice, Poland, were analysed in accordance with the Declaration of Helsinki. A new method has been proposed for the analysis of corneal deflection images in the Corvis tonometer with the use of the Canny edge detection method, mathematical morphology methods and context-free operations.

**Results:**

The resulting image analysis tool allows determination of the response of the cornea and the entire eyeball to an air puff. The paper presents the method that enables the measurement of the amplitude of curvature changes in the frequency range from 150 to 500 Hz and automatic designation of the eyeball movement direction. The analysis of these data resulted in 3 new features of dynamics of the eye reaction to an air puff. Classification of these features enabled to propose 4 classes of deformation. The proposed algorithm allows to obtain reproducible results fully automatically at a time of 5 s per patient using the Core i5 CPU M460 @ 2.5GHz 4GB of RAM.

**Conclusions:**

The paper presents the possibility of using a profiled algorithm of image analysis, proposed by the authors, to measure additional cornea deformation parameters. The new tool enables automatic measurement of the additional new parameters when using the Corvis tonometer. A detailed clinical examination based on this method will be presented in subsequent papers.

## Introduction

**Figure 1:**
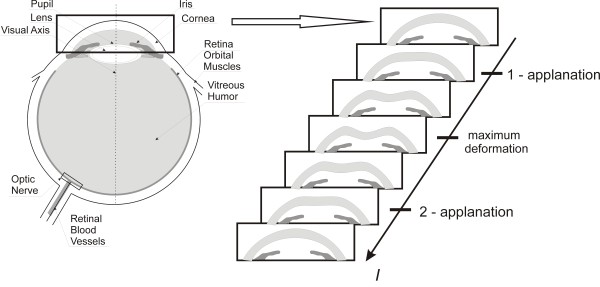
**Schematic diagram showing methodology of the measurement method and corneal response.** The cornea is subjected to an air-puff stimulus in the Corvis tonometer. In consequence, an image of the corneal deflection in the line arranged on the main axis is created every 23 μs. Individual deflection phases of greater importance are shown on the right.

**Figure 2:**
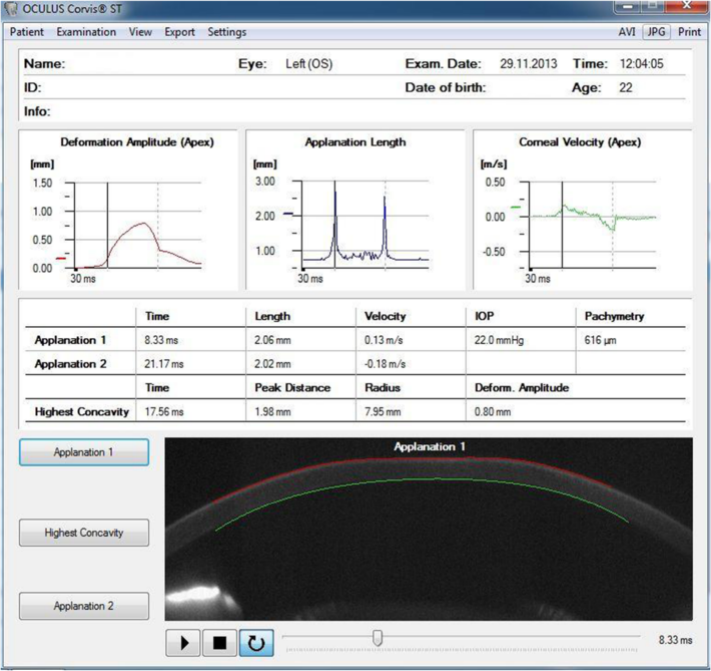
**Examples of results obtained from the Corvis device.** There are three graphs: deformation amplitude, applanation length, corneal velocity and two examples of applanation. For these two examples (1 and 2), time, length and velocity are given. On this basis and after linear calibration, the value of intraocular pressure IOP is determined.

Contemporary ophthalmology knows many methods of measuring intraocular pressure. These include non-contact and impression applanation tonometry. They are based on the Imbert-Fick law. Knowing the physical strength necessary to flatten a sphere and the surface of the flattening, it is possible to determine the pressure inside the sphere. Non-contact applanation tonometers, e.g. the Corvis, use this rule. In this tonometer, the flattening of the cornea is caused by an air puff - Figure 1. The device measures the cornea thickness, deflection amplitude, applanation length, corneal deflection speed, intraocular pressure. In addition, the information about places of applanation, pachymetry and other data are provided - Figure 2. The locations of applanation (temporary cornea flattening during a convex-concave transition and vice versa) enable to determine two applanation times *t*_1_ and *t*_2_. In Ocular Response Analyzer (ORA) the air pressure values *P*_1_ and *P*_2_ are read in applanation times *t*_*1*_ and *t*_*2*_, and then the value (*P*_1_ + *P*_2_)/2 is calculated. This value after linear calibration is the value of the intraocular pressure IOP. The Corvis tonometer also records sequence of images of the corneal deflection (resulting from the air puff). The sequence of images enables to get much more information using the methods of image analysis and processing. Such analysis allows to obtain three-dimensional reconstruction of corneal deflection in one of time ranges. On this basis, it is possible to determine the locations of applanation, the corneal deformation rate or the entire eyeball response to the air puff.

Analysis of images from the Corvis tonometer in the original software is based on designation of the outer and inner edges of the cornea profile. The edge detection methods of Roberts, Sobel and Prewitt can be all applied here. The Canny filter or local thresholding dependent on local brightness changes can be also used with equally good results. Additionally, the visible cross-sections of the iris which are often the brightest visible area are problematic here. All these methods based on a typical standard approach, enable to detect the corneal deformation in only one selected place (usually the main axis of fixation). From a biomechanical point of view, other locations of the corneal deformation or determination of the eyeball reaction to the air puff are also of interest. These parameters are not adequately described in the known publications in terms of both image analysis and processing as well as biomechanics.

The papers published so far are related to the analysis of corneal biomechanical parameters for the data derived mainly from the ORA device [1,2]. In [1], measurements of several populations indicate that corneal hysteresis, a biomechanical measure, varied over a dynamic range of 1.8 to 14.6 mmHg and was only weakly correlated with corneal thickness (*r*^*2*^ = 0.12). This is related to the observation that some subjects with relatively thick corneas have less-than-average corneal hysteresis. For the cornea itself, the effect of the patient's age [3-6], glaucoma [7] or wound healing [8] on its biomechanical parameters is also considered. Predictive numerical simulation of corneal biomechanical parameters was considered in an equally interesting way in [9]. In this paper, twenty-four corneal buttons were tested under posterior inflation conditions while monitoring their behaviour using non-contact methods. The measurement methods of the ultrastructure of the corneal stroma were presented in [10]. There are also very interesting studies profiled to the cornea analysis of Brazilian patients [11] or children with the use of the ORA tonometer [12,13]. A group of authors in [14] presented a correlation between hysteresis generated in a tonometer and pachymetry. By contrast, in [15] the same hysteresis as in [16] was presented in correlation with the glaucoma damage. The paper [17] deals with these issues (hysteresis), but in the Reichert ORA device. There exists also a well-known group of papers concerning the analysis of biomechanical parameters of the cornea in keratoconic eyes [18] or LASIK [19]. Moreover, there are descriptions of other tests of the cornea which contribute to the understanding of the biomechanical properties such as the comparison of the corneal strip extensometry presented in [20].

Last year has appeared series of papers dealing with application of Corvis noncontact tonometer to examine both IOP value as well as biomechanical parameters of the cornea, related to the corneal deformation [21-24]. However, Corvis tonometer gives more possibilities of corneal measurements after application of additional algorithms and procedures of data processing.

Therefore, there is a need to propose a dedicated automatic algorithm enabling measurements performed at out of axis places of the corneal deformation or determination of the eyeball response to an air puff during pressure measurement. The proposed dedicated algorithm and the results obtained from its use are discussed in detail later in the paper.

## Material

Images with an *M* × *N* × *I* resolution of 200 × 576 × 140 pixels were acquired from the Corvis device in the source recording format *.cst (they can be converted to a sequence of images *.bmp, *.jpg or a film *.avi). The patients ranging from 17 to 63 years of age were healthy (32 people including 16 women) and ill (16 people including 9 women). The group of ill patients suffered from either AMD or other diseases that cause abnormal pressure in the eye. A total of 96 eyes were examined and for each one a sequence of 140 images was obtained - which accounted for 13'400 2D images for analysis. The patients were examined during tests performed routinely in the Clinical Research Department of Ophthalmology, in District Railway Hospital in Katowice, Poland, in accordance with the Declaration of Helsinki. All the patient data have been anonymised.

## Method

### Pre-processing

Pre-processing of input images *L*_*GRAY*_(*m,n,i*), where *m*-row *m*∈(1,*M*), *n*-column *n*∈(1,*N*), *i* – another 2D image *i*∈(1,*I*), is related to their automatic reading from the source file with the extension *.cst. In the file, *.cst is a sequence of 2D images preceded by a header which contains basic information about the patient. A sequence of images *L*_*GRAY*_(*m,n,i*) thus obtained is then subjected to median filtering with a mask *h*_*1*_ sized *M*_*h1*_ × *N*_*h1*_ *× I*_*h1*_ = 3 × 3 × 3 pixels. The mask size was chosen arbitrarily, taking into account the size of distortions and artefacts entering the optical path. In case of larger mask size *h*_*1,*_ median filtering was causing the deletion of the corneal contour, therefore the size *M*_*h1*_ × *N*_*h1*_ *× I*_*h1*_ = 3 × 3 × 3 pixels was optimal. The filtered image *L*_*M*_(*m,n,i*) undergoes further preliminary transformations. These include designation of the outer edge of the cornea. Two different independent approaches to determining the outer limit of the cornea were implemented.

**The first one** involves analysis of brightness for each column of the image *L*_*M*_(*m,n = const,i = const*). Each column is subjected to binarization operation with a threshold set automatically according to the Otsu formula [25], i.e.:

(1) $$L_{B}\left(m,n,i\right)=\left\{\begin{array}{ccc} 1 & \mathrm{if} & L_{M}\left(m,n,i\right)>\left(v_{r}∙p_{r}\left(n,i\right)\right)\\ 0 & \mathrm{other} & \; \end{array}\right)$$

where: *p*_*r*_(*n*,*i*) - binarization threshold set automatically according to the Otsu formula for *m*∈(1,*M*),

*v*_*r*_ - automatic correction factor chosen arbitrarily at 0.5.

According to formula (1) binarization threshold is determined on the basis of two values: *p*_*r*_(*n,i*) which is the binarization threshold according to the Otsu and *v*_*r*_ which was chosen arbitrarily. Factor *v*_*r*_ is the correction factor resulting from the image specificity *L*_*M*_(*m,n,i*). There is a necessity of the correction of the threshold value *p*_*r*_(*n,i*) by 50% (0.5) due to high contrast of the corneal edges occurring in some cases. The resulting binary image *L*_*B*_(*m,n,i*) is analysed to enable determination of the outer edge of the cornea. One of the simplest methods is the calculation of the XOR value between two images. *L*_*B*_(*m,n,i*) is one image and the other one is the result of its morphological erosion *L*_*E*_(*m,n,i*) = min(*L*_*B*_(*m,n,i*)) with a structural element *SE* sized *M*_*SE*_ × *N*_*SE*_ × *I*_*SE*_ = 3 × 3 × 3 pixels. It results in the matrix *L*_*K*_(*m,n,i*):

(2) $$L_{K}\left(m,n,i\right)=\left(\min_{\mathrm{SE}}\left(L_{B}\left(m,n,i\right)\right)\right)\underline{\vee }\left(L_{B}\left(m,n,i\right)\right)$$

Since only the outer limit is determined, its contour *L*_*P*_^*(1)*^(*n*,*i*) can be defined as follows:

(3) $$L_{R}\left(m,n,i\right)=\left\{\begin{array}{ccc} m & \mathrm{if} & L_{K}\left(m,n,i\right)=1\\ 0 & \mathrm{other} & \; \end{array}\right)$$

(4) $$L_{P}^{\left(1\right)}\left(n,i\right)=\left\{\begin{array}{ccc} \min_{m}\left(L_{R}\left(m,n,i\right)\right) & \mathrm{if} & L_{K}\left(m,n,i\right)=1\\ 0 & \mathrm{other} & \; \end{array}\right)$$

The results are, to a large extent, influenced by the brightness of the iris cross-section which, in turn, affects the overvaluation of the threshold value *p*_*r*_. As a result, the proper edge of the cornea is not properly recognized. For the analysed 13'400 2D images, the above method of edge detection works well in about 80% of cases. The value of 80% of cases was defined as the number of correctly detected corneal contours in all analyzed cases (13'400 2D images). The correctly detected corneal contour was described as not having any discontinuity points and the difference of the position of each contour point was below 10 pixels (maximum displacement of the contour line) between following series images.

**The other approach** involves using the well-known Canny edge detection method. The obtained result *L*_*C*_(*m,n,i*) of edge detection, for the arbitrarily selected parameters of the threshold (0.1) and standard deviation of the Gaussian filter (0.99), requires further correction. This adjustment only involves morphological closing, i.e.:

(5) $$L_{W}\left(m,n,i\right)=\min_{\mathrm{SE}}\left(\max_{\mathrm{SE}}\left(L_{C}\left(m,n,i\right)\right)\right)$$

Next, the binary image *L*_*W*_(*m,n,i*) is subjected to determination of the upper edge analogously to (3), (4). The resulting contour of the outer edge of the cornea *L*_*P*_^*(2)*^(*n,i*) is correct in about 90% of cases for the analysed 13'400 2D images. The definition of the correctly detected corneal contours were analogous to the previously described method.

**Figure 3:**
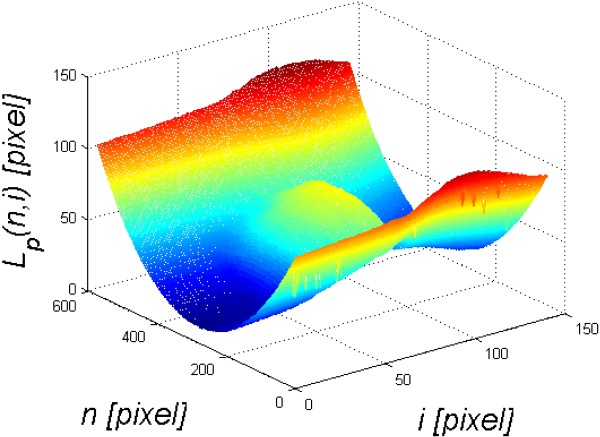
**Example of a cornea outer edge detection result.** The image *L*_*P*_(*n,i*) results from the Canny edge analysis and additional processing proposed by the authors. Local problems with edge detection can be removed here by median filtration with a mask sized 3 × 3 pixels. This image is further processed.

Incorrectly detected cornea outer edges coincide for both methods, (*L*_*P*_^*(1)*^(*n,i*)) and *L*_*P*_^*(2)*^(*n,i*)), and for this reason they cannot be mutually complementary. Due to better edge detection in the case of the second method (Canny), it was used further (*L*_*P*_(*n,i*) for simplification). The results are shown in Figure 3.

### Processing

The outer edge of the cornea *L*_*P*_(*n,i*), determined in image pre-processing, forms the basis for further analysis. Automatic designation of two important parameters, namely the correction of the cornea deformation curve and the rapidly changing cornea deformation in dynamic states when applying force, is described below.

### Cornea deformation correction

Under the influence of a pressure impulse from the Corvis tonometer, the cornea is deformed and the entire eyeball moves in the eye socket. For proper evaluation and interpretation of the results, displacement of the entire eyeball should be separated from the deflection curve of the cornea. For this purpose, in the first step, the shape of the cornea visible in time *t* = 0 (for *i* = 1) is removed, i.e.:

(6) $$L_{T}\left(n,i\right)=L_{P}\left(n,i\right)-L_{P}\left(n,1\right)$$

**Figure 4:**
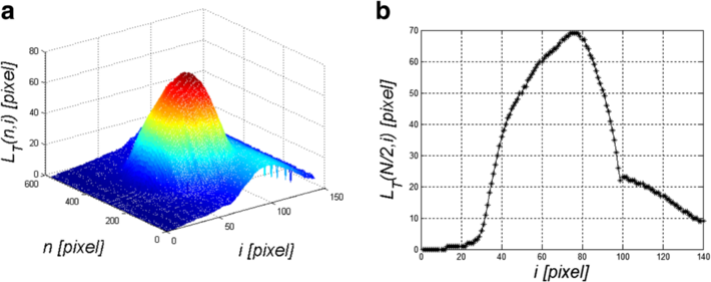
**Sample graphs *L***_***T***_**( *n,i *) and *L***_***T***_**( *N/2,i *).** The results obtained concern a relative cornea deformation resulting from an air-puff stimulus. The cross-section along the main axis *N*/2 is shown on the left **a)**. This graph consists of two overlapping waveforms. One refers to the cornea deformation and the other one to the entire eyeball response to an air puff. The object of the analysis is, inter alia, separation of these two waveforms - **b)**.

The resulting image *L*_*T*_(*n,i*) and the graph *L*_*T*_(*N/2,i*) are shown in Figure 4. The graph *L*_*T*_(*N/2,i*) consists of two parts of the response: corneal deflection *L*_*TR*_(*N/2,i*) and the eyeball reaction *L*_*TO*_(*N/2,i*) to an air-puff stimulus, in general:

(7) $$L_{T}\left(n,i\right)=L_{\mathrm{TO}}\left(n,i\right)+L_{\mathrm{TR}}\left(n,i\right)$$

The separation of these two waveforms (*L*_*TR*_(*N/2,i*) and *L*_*TO*_(*N/2,i*)) from *L*_*T*_(*N/2,i*) is possible owing to the analysis of the visible contour of the cornea on the left and right borders of the image, i.e.:

(8) $$L_{\mathrm{TO}}\left(n,i\right)=\frac{L_{T}\left(N,i\right)-L_{T}\left(1,i\right)}{N-1}∙\left(n-1\right)+L_{T}\left(1,i\right)$$

**Figure 5:**
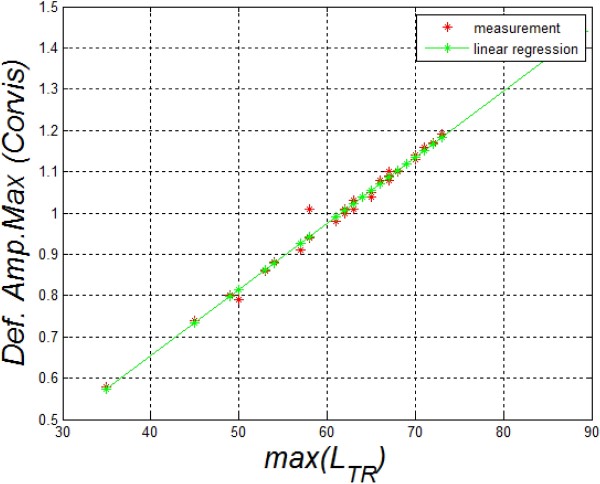
**Correlation between the maximum amplitude of the cornea deformation calculated with the Corvis software and the author’s algorithm.** These results confirm the correctness of the adopted algorithm of analysis and processing of image sequences and their reading from the format *.cst. On this basis, other parameters described in the paper were further determined and calculated.

**Figure 6:**
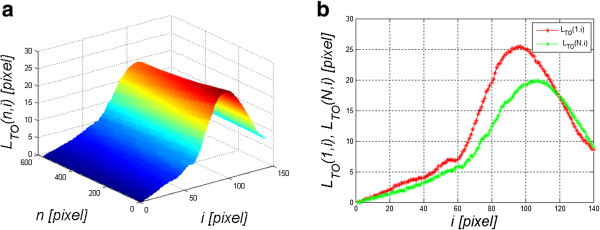
**Examples of analysis results - the image *L***_***TO***_**(*n,i*) is the eyeball displacement.** Additionally, the graph on the right **a)** shows the waveforms *L*_*TO*_(*1,i*) and *L*_*TO*_(*N,i*). Using the methods of image analysis and processing described by the authors, the proper corneal deflection caused by an air puff can be separated from the eyeball displacement - **b)**.

**Figure 7:**
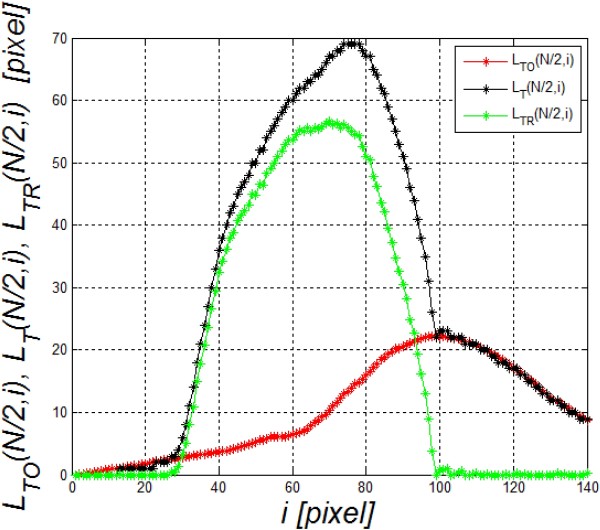
**Waveform *L***_***T***_**(*n,i*), *L***_***TO***_**(*n,i*) and *L***_***TR***_**(*n,i*).** Separation of the waveforms (*L*_*TO*_(*n,i*) and *L*_*TR*_(*n,i*)) enabled separation of the entire eyeball reaction from the cornea response to an air-puff stimulus. Therefore, further analysis and measurement of properties specific for each of them is possible.

The waveform *L*_*TO*_(*n,i*) at the point *N*/2 defined with the formula (8) can be subtracted from *L*_*T*_(*N/2,i*) (formula (7)). The correctness of the algorithm was pre-verified by determining the correlation between the maximum amplitude of deviation calculated by the Corvis software and the same quantity determined from the equation (7) - *max*(*L*_*TR*_). The results obtained are shown in Figure 5 - the error calculated as the mean standard deviation is less than 1%. Next, image analysis results are shown in Figure 6. These results (Figure 6) are extremely interesting from the point of view of biomechanics of the eye. The movement of the eyeball in response to a pressure impulse can be observed. As a result, it is possible to correct the waveform of the cornea deformation itself. Also the symmetry in the waveforms *L*_*TR*_(*N/2,i*) and *L*_*TO*_(*N/2,i*) is quite clearly visible - Figure 7. Separation of the eyeball displacement *L*_*TO*_(*n,i*) from the waveform of the cornea deformation *L*_*T*_(*n,i*) allows to measure another new parameter - rapidly changing cornea deformations visible during force application.

### Rapidly changing cornea deformations during force application

Rapidly changing cornea deformations (with a frequency above 150 Hz) are directly visible in Figure 4. These deformations are not measured by the Corvis tonometer and they contain significant information about the biomechanical parameters of the eye (in particular the cornea). Their measurement is possible owing to the profiled image analysis algorithm.

**Figure 8:**
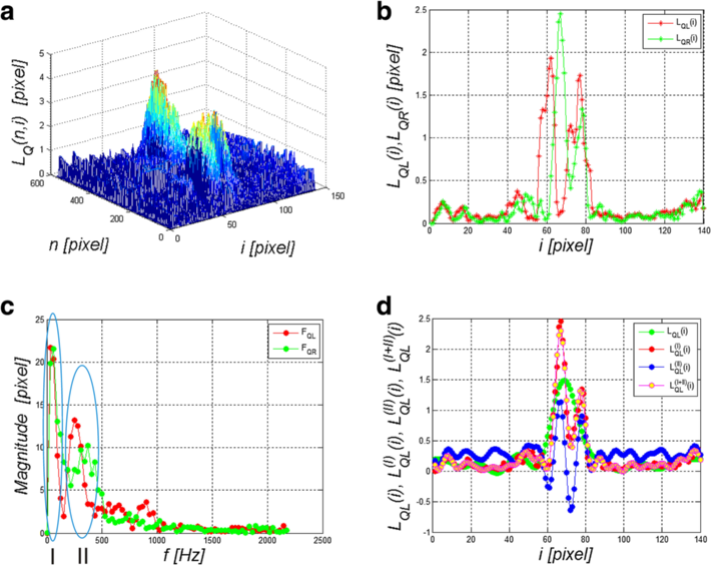
**Sample result - the image *L***_***Q***_**(*n,i*) and its frequency analysis.** Figure **a)** shows *L*_*Q*_(*n,i*), **b)** shows graphs *L*_*QL*_(*i*) and *L*_*QR*_(*i*), **c)** shows frequency analysis *F*_*QR*_ and *F*_*QL*_ of the graphs *L*_*QL*_(*i*) and *L*_*QR*_(*i*), **d)** shows individual components *L*_*QL*_^*(I)*^(*i*) and *L*_*QL*_^*(II)*^(*i*) and their sum *L*_*QL*_^*(I+II)*^(*i*) of the waveform *L*_*QL*_(*i*).

The image *L*_*TR*_(*n,i*), resulting from the previous processing stage, undergoes an operation which involves the removal of a slowly changing deformation. For this purpose, the image resulting from morphological opening is subtracted from the image *L*_*TR*_(*n,i*), i.e.:

(9) $$L_{Q}\left(n,i\right)=L_{\mathrm{TR}}\left(n,i\right)-\max_{\mathrm{SE}2}\left(\min_{\mathrm{SE}2}\left(L_{\mathrm{TR}}\left(n,i\right)\right)\right)$$

for a structural element *SE2* sized *N*_*SE2*_ × *I*_*SE2*_ = 33 × 33 pixels. The size of the structural element *SE2* is matched to the low-frequency range, lasting more than a few dozen milliseconds. The resulting image *L*_*Q*_(*n,i*) is shown in Figure 8a, whereas Figure 8b presents the graphs *L*_*QL*_(*i*) and *L*_*QR*_(*i*). The results of FFT (Fast Fourier Transform), namely *F*_*QR*_ and *F*_*QL*_, of the graphs *L*_*QL*_(*i*) and *L*_*QR*_(*i*) are shown in Figure 8c, whereas Figure 8d presents individual components *L*_*QL*_^*(I)*^(*i*) and *L*_*QL*_^*(II)*^(*i*) as well as their sum *L*_*QL*_^*(I+II)*^(*i*) of the waveform *L*_*QL*_(*i*). The individual components *L*_*QL*_^*(I)*^(*i*) and *L*_*QL*_^*(II)*^(*i*) were obtained from harmonics marked in Figure 8c with areas I and II. These are respectively: I- constant and slowly changing component (below 150 Hz) and II- component responsible for periodic rapidly changing (after the above 200 Hz) positions of the cornea on the left side relative to the axis of symmetry (*L*_*QL*_(*i*)). The analysis of the graphs *F*_*QR*_ and *F*_*QL*_ (Figure 8c) provides very valuable information regarding the frequency range of the cornea changes under the influence of an air puff. It should be noted that the frequency range for the analysed cases of 96 eyes is 150 to 500 Hz.

**Figure 9:**
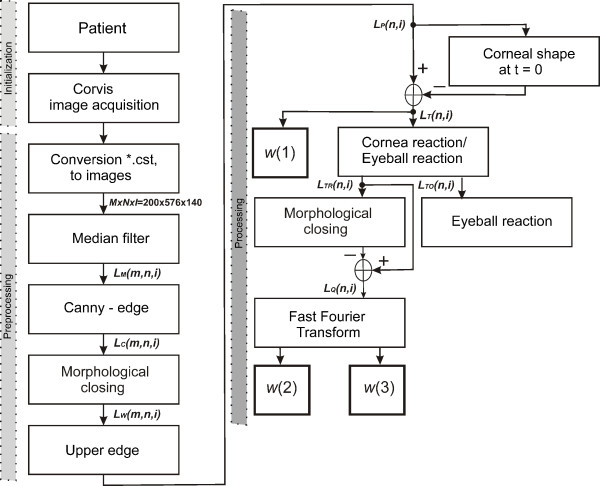
**Block diagram of the proposed image analysis algorithm.** These are, in order, image acquisition from the Corvis tonometer, median filtration, determination of the cornea outer edge, separation of the relative cornea deformation, separation of reactions, eyeball displacement and rapidly changing cornea deformation. As a result, new indicators describing the biomechanical parameters of the eye were obtained during force application with a pressure impulse.

A block diagram of the proposed algorithm is shown in Figure 9.

## Results

On the basis of the proposed algorithm, a pattern *L*_*V*_(*n,i*) of the response of the cornea and the entire eyeball to an air puff was created:

(10) $$L_{V}\left(n,i\right)=L_{\mathrm{TO}}\left(N/2,i\right)+\max_{\mathrm{SE}2}\left(\min_{\mathrm{SE}2}\left(L_{\mathrm{TR}}\left(n,i\right)\right)\right)$$

The obtained images of 96 eyes were compared to the pattern created for each eye separately:

(11) $$L_{\Delta }\left(n,i\right)=L_{T}\left(n,i\right)-L_{V}\left(n,i\right)$$

**Figure 10:**
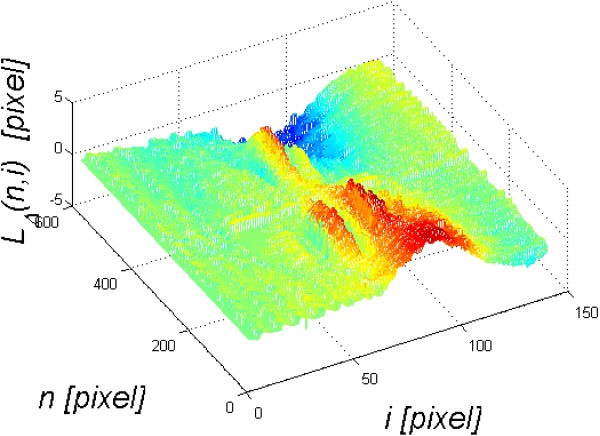
**Sample result of the comparison of the registered waveform *L***_***T***_**(*n,i*) with the pattern *L***_***V***_**(*n,i*).** The resulting error value *L*_*Δ*_(*n,i*) is shown above. The rapidly changing cornea deformation during force application and the change in the eyeball position in the eye socket in response to an air-puff stimulus are clearly visible.

A sample result, the error *L*_*Δ*_(*n,i*), is shown in Figure 10. *L*_*Δ*_(*n,i*) is the basis for further analysis. Based on *L*_*Δ*_(*n,i*), the following new features are calculated:

*w*(1) - the maximum amplitude of the eyeball deflection during force application:

(12) $$w\left(1\right)=\max_{i\in \left(1,I\right),n\in \left\{1,N\right\}}\left(L_{\Delta }\left(n,i\right)\right)$$

*w*(2) - the maximum amplitude in the spectra *F*_*QR*_ and *F*_*QL*_ for the frequency range from 150 to 500 Hz. With a fixed number of frames per second during registration with the Corvis tonometer (one frame every 23 μs), it is:

(13) $$w\left(2\right)=\max_{i\in \left(7,15\right)}\left(F_{\mathrm{QR}}\left(i\right),F_{\mathrm{QL}}\left(i\right)\right)$$

*w*(3) - the duration of the rapidly changing (from 150 to 500 Hz) cornea deformations calculated as a time range with a threshold of 80% of the maximum amplitude - Figure 10.

**Figure 11:**
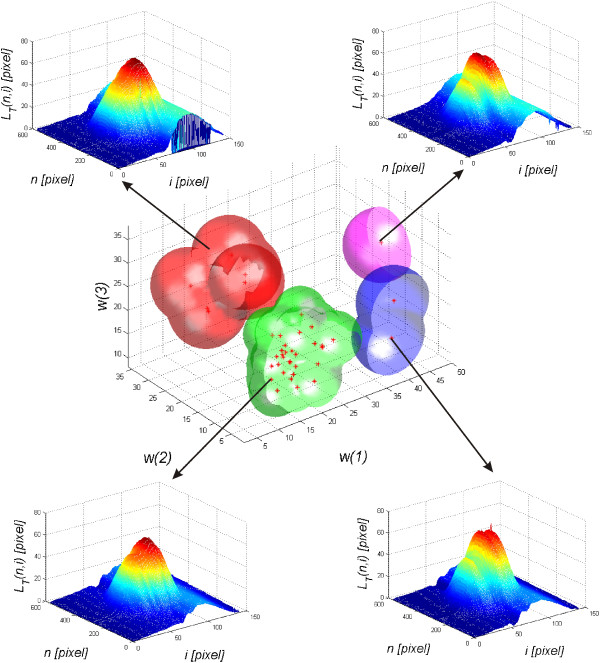
**Results of the correlation of the features *w*(1) and *w*(2) with *w*(3).** The graph shows changes in the features *w*(1) and *w*(2) with *w*(3) measured with the proposed algorithm. The graph also shows 4 characteristic clusters (classes) marked in red, green, blue and magenta. The characteristic waveforms *L*_*T*_(*n,i*) corresponding to the classes 1,2,3,4 are shown in the corners.

**Figure 12:**
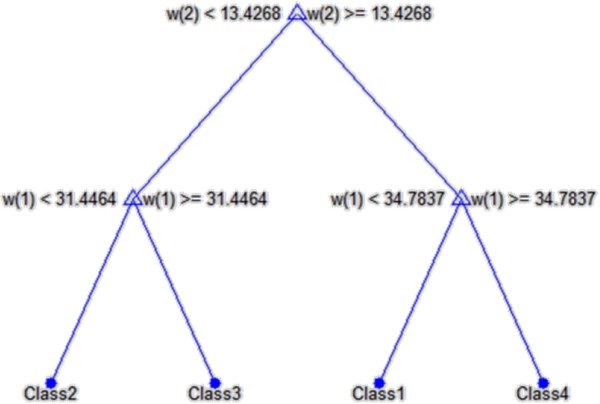
**Decision tree created on the basis of the features *w*(1), *w*(2), *w*(3) acquired from the image and their division into four classes.** Due to good separation of individual classes and a small number of nodes, the created decision tree requires no pruning (there is no concern that the decision tree will be overfit to the data).

**Figure 13:**
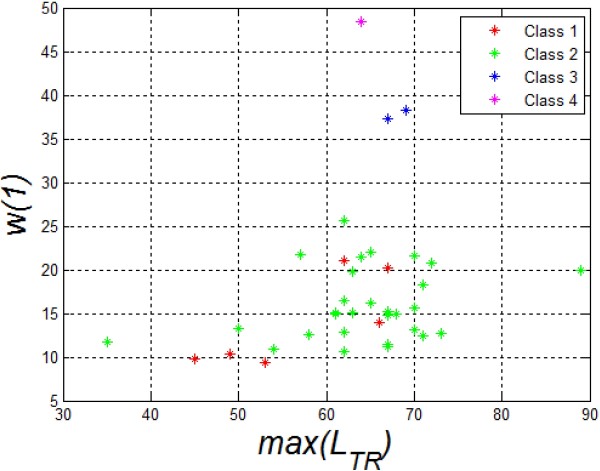
**Graph showing the dependence of the maximum eyeball displacement *w*(1) on the cornea deformation *max*(*L***_***TR***_**) for each class.** The graph results from the division of deformations automatically measured with the proposed algorithm into 4 classes. The results obtained provide the basis for further analysis and comparison of biomechanical parameters of the eye in future studies.

The choice of features and their number is closely related to the previously described automatically calculated parameters such as the correction of the cornea deformation or the entire eyeball reaction to a stimulus. The obtained results, the values of *w*(1), *w*(2) and *w*(3), measured with the Corvis tonometer for the first 10 eyes are shown in Table 1. The correlation results of the features *w*(1) and *w*(2) with *w*(3) are shown in Figure 11. The results presented in Figure 11 were divided into 4 different classes. The adopted number of classes is the result of the analysis of the distribution of individual clusters forming sets that do not have common features. On this basis, a decision tree was created which is shown in Figure 12. In all cases, a non-parametrical algorithm CART (Classification and Regression Trees) creating binary trees is used as the method for their induction. An increase in the nodes purity has been used as the criterion for assessing the quality of CART divisions. The Gini index has been used as the measure of nodes impurity. Because of a small number of cases, the tree creation was not limited by a minimum number of vectors in a node. In its current form (Figure 12), there is no need to trim the tree – there is no concern that the decision tree will be overfit to the data. For the analysed number of cases, all classification results are correct. The number of the eyes classified as Class 1 is 6 (Figure 11 - red), Class 3–2 (Figure 11 blue), Class 4 – 2 (Figure 11 magenta), and the rest belongs to Class 2 (Figure 11 green). An increase in the number of the analysed eyes (patients) may, in the future, give more reliable results and provide additional evidence as to the number of classes. The individual classes, depending on the range of feature values (*w*(1), *w*(2) and *w*(3)) which is characteristic for them, define various types of reactions of the entire eyeball to a stimulus and different amplitudes of rapidly changing deformations of the cornea itself. A sample graph of the dependence of the maximum displacement of the entire eyeball *w*(1) on the cornea deformation *max*(*L*_*TR*_) for each class is shown in Figure 13. Both the features as well as the division into classes can be further used not only as an additional diagnostic tool but they can also enrich the existing software of the Corvis tonometer.

**Table 1:** **Summary of sample features *w*** **(1), *w*** **(2) and *w*** **(3) for 10 eyes (only the right ones) and their division into classes according to the proposed classifier**

**No**	***w*** **(1)**	***w*** **(2)**	***w*** **(3)**	**Class**
**1**	37.2	2.5	17.0	1
**2**	15.0	3.2	16.8	2
**3**	13.1	4.3	22.0	2
**4**	25.6	5.0	19.5	2
**5**	20.2	28.6	28.6	3
**6**	13.9	16.3	30.6	3
**7**	38.2	3.2	24.2	1
**8**	48.4	20.3	25.1	4
**9**	21.0	25.5	25.6	3
**10**	20.7	5.2	23.9	2

### Critical summary

The paper presents the possibility of using the profiled algorithm of image analysis, proposed by the authors, to measure additional parameters of the corneal deformation. This algorithm enables separation of the eyeball reaction from the corneal deflection during force application. In addition, measurements of rapidly changing cornea deformations (from 150 to 500 Hz) were proposed. These measurements allowed to propose a pattern individually for each eye. The pattern, in turn, helped to determine deviations individually for each eye. On the basis of these deviations, the features *w*(1), *w*(2) and *w*(3) as well as the classifier (decision tree) allowing a division into four classes were calculated.

The paper only presents a tool for automatic measurement of additional new parameters when using the Corvis tonometer. A detailed clinical examination based on this method will be presented in subsequent papers. In addition, the accuracy and repeatability of measurements for one patient and a phantom, the correlation of features (primarily *w*(1)) for a single patient and the right and left eyes and the effect of the patient position during the test on the result, both IOP as well as the other features, will be verified. The issue of analysis of symmetry, or its lack, in the dynamic response of the entire eyeball to an air-puff stimulus, is quite interesting and remains open. The analysis of corneal deflection parallel in other cross-sections is an open topic here. From a practical point of view, higher harmonics (above 2 kHz), that for the current sampling frequency (every 23 μs) are difficult to analyse, are also interesting. Moreover, the presented methods of image analysis and processing may be carried out in another way, in another dedicated algorithm, for example, with the use of random methods of analysis of layers in tomographic eye images [26,27], the methods of mathematical morphology [28] or others [29]. The final choice of the analysis method is highly dependent on the results obtained in practice, which is an important element given the large inter-individual variability. Indeed, biomechanical parameters of the cornea, measured in dynamic states, still represent a new area of interdisciplinary research.

## Abbreviations

ROI: Region Of Interest; IOP: Intraocular Pressure; FFT: Fast Fourier Transform; LASIK: Laser-Assisted in situ Keratomileusis.

## Competing interests

The authors declare that they have no competing interests.

## Authors’ contributions

RK suggested the algorithm for image analysis and processing, implemented it and analysed the images, wrote the full text of the manuscript. AL, AN, EW, ZW performed the acquisition the images from Corvis and HK consulted the obtained results. All authors have read and approved the final manuscript.
